# Interpreting vascular smooth muscle responses to cyclic stretch and hydrostatic pressure

**DOI:** 10.1016/j.jphyss.2026.100117

**Published:** 2026-10-23

**Authors:** Marie Frances S. Villegas

**Affiliations:** Saint Joseph College of Baggao, San Jose, Baggao, Cagayan, Philippines

**Keywords:** Vascular smooth muscle cells, Cyclic stretch, Hydrostatic pressure, Mechanical stress, Vascular remodeling, Hypertension

We read with interest the study by Kusume et al. [1], which compared transcriptomic and secretomic responses of human aortic smooth muscle cells to cyclic stretch (10%) and cyclic hydrostatic pressure corresponding to normotensive (120/60 mmHg) and hypertensive (180/120 mmHg) conditions. The distinct molecular profile observed under hypertensive pressure offers a useful starting point for understanding vascular mechanotransduction. Three considerations may help clarify its physiological interpretation and guide subsequent investigations.

First, the comparison of cyclic stretch with hydrostatic pressure raises an important question about the mechanical context of the findings. In the arterial wall, circumferential deformation, transmural pressure, matrix stiffness, and shear-related signals coexist and interact. Distinct molecular signatures in isolated loading conditions therefore demonstrate stimulus-specific cellular responses, but cannot alone establish how these signals combine in intact vessels. Experiments that systematically vary pressure and stretch together, while documenting deformation and substrate properties, could distinguish pressure-sensitive signaling from responses secondary to changes in cell strain or the extracellular matrix.

Second, the marked upregulation of vasoactive intestinal peptide receptor 1 (VIPR1) under hypertensive hydrostatic pressure is intriguing but should be interpreted as a molecular observation rather than evidence of a particular functional vascular outcome. Transcript abundance does not necessarily predict receptor protein abundance, membrane localization, or downstream signaling. Follow-up studies could measure VIPR1 protein and receptor-mediated signaling and examine whether pharmacological inhibition or gene silencing alters contractile markers, calcium handling, proliferation, or extracellular-matrix production. Such experiments would help determine whether VIPR1 induction reflects compensation, maladaptation, or a response without a direct functional consequence.

Third, temporal and biological heterogeneity merit attention. Aortic smooth muscle cells may exhibit early stress signaling followed by later phenotypic remodeling, and responses can differ with donor characteristics, passage number, and baseline contractile phenotype. Time-course experiments, biological replication across donors, and validation in intact vascular tissue could help separate transient adaptation from persistent remodeling. Integrating transcriptomic and secretomic changes with measurable physiological endpoints—such as force generation or vessel stiffness—would further strengthen translation from cell culture to vascular function.

The study by Kusume et al. [1] makes a valuable contribution by showing that hypertensive hydrostatic pressure cannot be treated as interchangeable with cyclic stretch. Its greatest promise may lie in motivating mechanistically controlled studies that connect stimulus-specific molecular profiles with causal pathways and demonstrable vascular phenotypes. Such work could sharpen our understanding of how mechanical loading contributes to vascular adaptation and dysfunction in hypertension.

## CRediT authorship contribution statement

**Marie Frances S. Villegas:** Conceptualization, Writing – original draft, Validation.

## Consent for publication

Not applicable.

## Ethics approval and consent to participate

Not applicable; this letter reports no original research involving human participants or animals.

## Funding

No funding was received for preparation of this letter.

## Competing interests

The author declares no competing interests.

## Declaration of Competing Interest

The authors declare that they have no known competing financial interests or personal relationships that could have appeared to influence the work reported in this paper.

## Data Availability

Not applicable; no new datasets were generated or analyzed.
